# Hematopoietic stem cell transplantation for children with *β*-thalassemia major: multicenter experience in China

**DOI:** 10.1007/s12519-017-0107-5

**Published:** 2018-03-06

**Authors:** Xin-Yu Li, Xin Sun, Jing Chen, Mao-Quan Qin, Zuo Luan, Yi-Ping Zhu, Jian-Pei Fang

**Affiliations:** 1 Guangdong Provincial Key Laboratory of Malignant Tumor Epigenetics and Gene Regulation, Medical Research Center (Pediatrics), Medical Research Center, Sun Yat-Sen Memorial Hospital, Sun Yat-Sen University, Guangzhou, China; 2 Department of Hematology, Guangzhou Women and Children’s Medical Center, Guangzhou Medical University, Guangzhou, China; 3 Department of Hematology, Shanghai Children’s Medical Center, Shanghai Jiaotong University School of Medicine, Shanghai, China; 4 Department of Hematology, Beijing Children’s Hospital, Capital Medical University, Beijing, China; 5 Department of Pediatrics, Navy General Hospital PLA China, Beijing, China; 6 Department of Pediatrics, West China Second University Hospital/West China Women and Children’s Hospital, Sichuan University, Chengdu, China; 7 Department of Pediatrics, Sun Yat-Sen Memorial Hospital of Sun Yat-Sen University, No. 107, West Yan Jiang Road, Guangzhou, 510120 China

**Keywords:** *β*-Thalassemia major, Hematopoietic stem cell transplantation, Umbilical cord blood

## Abstract

**Background:**

*β*-Thalassemia major (*β*-TM) has become a public health problem in mainland China. Hematopoietic stem cell transplantation (HSCT) has remained the only cure for *β*-TM in mainland China since 1998.

**Methods:**

This multicenter retrospective study provides a comprehensive review of the outcomes of 50 pediatric patients with *β*-TM who received HSCT between 1998 and 2009 at five centers in mainland China. Both related (*n* = 35) and unrelated donors (*n* = 15) with complete human leukocyte antigen matches were included. The stem cell sources included bone marrow (BM), peripheral blood stem cells, umbilical cord blood (UCB) and a combination of BM and UCB or a combination of BM and peripheral blood stem cells from a single sibling donor.

**Results:**

The probabilities of 5-year overall survival (OS) and thalassemia-free survival (TFS) after the first HSCT were 83.1 and 67.3%, respectively. Graft failure (GF) occurred in 17 patients. Univariate analyses showed that umbilical cord blood transplantation (UCBT) was one of the potential risk factors for decreased OS (*P* = 0.051), and that UCBT (*P* = 0.002) was potentially related to TFS. GF incidence was distinct between the UCBT and non-UCBT groups (*P* = 0.004). Four cases of UCB-BM combined transplantation led to decreased risks of mortality and recurrence. In the UCBT group, related donor transplantation produced more favorable results than unrelated donor transplantation in OS (*P* = 0.009) but not in TFS (*P* = 0.217).

**Conclusions:**

GF was the primary cause of UCBT failure. Though UCBT from related donors was not favorable, the combined transplantation of UCB and BM could improve the prognosis of UCBT.

## Introduction

Thalassemia is the most common single-gene disorder worldwide and is considered a major public health issue. *β*-Thalassemia major (*β*-TM) occurs in homozygous or compound heterozygous states for *β*-hemoglobin gene mutations that either reduce (*β*^+^-thal) or abolish (*β*^0^-thal) expression of the affected *β*-globin genes [1]. The patients require long-term regular blood transfusions and chelation therapy, and children with untreated or partially treated *β*-TM die in the first or second decade of life [2]. *β*-TM has become a public health problem in mainland China. In the 1980s, a large-scale survey of hemoglobinopathies was carried out [3]. In recent years, 13,397 samples from Guangdong Province have been analyzed for both hematological and molecular parameters showing a high prevalence of carriers of *α*-thal (8.53%), *β*-thal (2.54%), and both *α*- and *β*-thal (0.26%) [4]. Hematopoietic stem cell transplantation (HSCT) has remained the only cure for *β*-TM [5] since the first bone marrow transplantation (BMT) for *β*-TM reported in December 1981 [6]. This expensive procedure was not successfully performed in mainland China until 1998 [7]. In China, many patients with *β*-TM cannot afford life-long regular blood transfusions and iron chelation. Although HSCT is expensive, it is a one-time treatment that is possible for some patients. Due to differences in medical, socioeconomic and cultural situations, there is wide variation in the treatment of *β*-TM between developed and developing countries. The disease-free survival rates after HSCT have ranged 52–82% in China [8–12]. The majority of patients with *β*-TM are distributed throughout southern China and HSCT is performed by several qualified hospitals. Each center has reported a few cases, though most are without systemic analysis [8–10, 12–15]. To provide a comprehensive review of the outcomes of children receiving HSCT for *β*-TM in China, the present study retrospectively analyzed the data from children receiving HSCT for *β*-TM between 1998 and 2009 in a multicenter study group of the Pediatric Branch of the Chinese Medical Association.

## Methods

### Patient characteristics

The study was approved by the Institutions’ Ethical Committee, and informed consent was obtained from the patients’ parents. The recipient and donor characteristics are summarized in Table 1. The study included patients with *β*-TM younger than 18 years at transplantation who received HSCT between January 1998 and December 2009 in mainland China. Fifty cases of first HSCT for genetically and symptomatically transfusion-dependent thalassemia were included in this retrospective study. Complete human leukocyte antigen (HLA) matches between 6 points of the -A, -B and -DR alleles were required. Patients were stratified according to the Pesaro risk factors [16, 17]: 37.0% of the patients were Pesaro class 3, 44.4% were class 2, and 18.5% were class 1.

**Table 1:** Recipient and donor characteristics (*n* = 50)

Variables	Values
Gender, *n* (%)	
Male	28 (56.0)
Female	22 (44.0)
Age at HSCT, *n* (%)	5.0 (1.0–14.0)
< 7 y	32 (64.0)
≥ 7 y	18 (36.0)
Pesaro class, *n* (%)	
1	5 (18.5)
2	12 (44.4)
3	10 (37.0)
NA	23
Donor type, *n* (%)	
Related	35 (70.0)
Unrelated	15 (30.0)
Graft type, *n* (%)	
PBSC	10 (20.0)
UCB	22 (44.0)
BM	9 (18.0)
BM + PBSC/UCB	9 (18.0)
BM + PBSC	5 (10.0)
BM + UCB	4 (8.0)
Conditioning regimen, *n* (%)	
BUCYATG	11 (22.0)
BUCYATG + Flu + Hu	14 (28.0)
BUCY + X	25 (50.0)
GVHD prophylaxis, *n* (%)	
CsA	16 (32.0)
CsA + MTX	21 (42.0)
CsA + MMF	5 (10.0)
CsA + MTX + MMF	6 (12.0)
CsA + MTX + zenepax	2 (4.0)

*HSCT* hematopoietic stem cell transplantation, *PBSC* peripheral blood stem cell, *UCB* umbilical cord blood, *BM* bone marrow, *BU* busulfan, *CY* cyclophosphamide, *ATG* anti-thymocyte globulins, *Flu* fludarabine, *Hu* hydroxyurea, *CsA* cyclosporine A, *MTX* short courses of methotrexate, *MMF* mycophenolate mofetil, *NA* not available, *BUCYATG* conditioning regimen consisting of BU, CY and ATG, *BUCYATG* *+* *Flu* *+* *Hu* conditioning regimen consisting of BU, CY and ATG, Flu, Hu and Aza, *BUCY* *+* *X* all other conditioning regimens

The stem cell sources included bone marrow, peripheral blood stem cells, umbilical cord blood (UCB) and a combination of cord blood/peripheral blood stem cells and bone marrow (BM) from a single sibling donor. If the sibling donor was too young to reach the criterion of 15 kg, combined transplantation was considered. Nucleated cell counts (NC) were 8.0–10.0 × 10^8^/kg recipient weight at the time of BMT or peripheral blood stem cell transplantation (PBSCT). NCs were 3.5–3.7 × 10^7^/kg recipient weight at umbilical cord blood transplantation (UCBT). The proportion of CD34^+^ was 0.5–1%.

### Transplantation procedures

Details regarding conditioning regimens and graft-versus-host disease (GVHD) prophylaxis are provided in Table 1. Patients received myeloablative BUCY-based conditioning regimens [intravenous busulfan (total 11.2–12.8 mg/kg, divided into 16 fractions from days − 9 to − 6) combined with cyclophosphamide (total 120–200 mg/kg, divided into 4 fractions between days − 5 and − 2)] without blood concentration monitoring. Forty-eight (96%) patients accepted anti-thymocyte globulins [ATG (horse ATG before 2001, total 90–100 mg/kg, 3 or 4 fractions, started at day − 4; rabbit ATG after 2001, total 7.5–11.5 mg/kg)]. Fludarabine (Flu), thiotepa (TT) or melphalan (Mel) were added to the myeloablative conditioning regimen to maximize the elimination of the recipients’ hematopoietic stem cells, especially in the extramedullary hematopoietic sites. Total body irradiation was seldom (2 of 50) applied for children with non-malignant conditions due to its growth-retarding effects and risk for secondary malignancies. Beginning in 2001, hydroxyurea (Hu, 30 mg/kg daily) and azathioprine (Aza, 3 mg/kg daily) were given together 3–4 weeks before busulfan conditioning. For GVHD prophylaxis, patients received cyclosporine A (CsA) starting at 2.5–3.0 mg/kg intravenously daily on day − 1 with a plasma concentration of 150 to 250 ng/mL or in combination with short courses of methotrexate (MTX, 15 mg/m^2^ on day 1 and 10 mg/m^2^ on days 3, 5 and 11), mycophenolate mofetil (MMF, 30 mg/kg/day divided into 2 fractions starting on day 1), methylprednisolone (1 mg/kg) or daclizumab (1 mg/kg on day − 1, repeated every 2 weeks; 5 doses in total). In the majority of patients (74.0%), GVHD prophylaxis consisted of CsA alone (*n* = 16) or a combination of CsA and short courses of MTX (*n* = 21). Fourteen of 22 cord blood recipients accepted GVHD prophylaxis that included CsA along. Five of the cord blood recipients accepted CsA together with MMF. Supportive therapy and the post-transplantation use of hematopoietic growth factors was in accordance with the policies of each individual center.

### Definition of end points

The endpoints of the primary study were thalassemia-free survival (TFS) and overall survival (OS). For the analysis of OS, failure was defined as death from any cause, and surviving patients were censored at the date of last contact. TFS was defined as survival without graft failure or a second transplantation. Graft failure (GF) was clinically defined as persistent pancytopenia with no hematological recovery or recurrent *β*-TM. Chimerism data were incomplete, and when available, were captured using different techniques over time (e.g., sex chromosome hybridization in situ, analysis of variable number tandem repeat polymorphisms, or microsatellite analysis).

### Statistical analyses

The characteristics of patients and transplantation were studied with descriptive analyses. Univariate comparisons were made using the Chi-square test or Fisher’s exact test for dichotomous variables and Pearson’s exact Chi-square test for qualitative variables of more than two categories. For continuous variables, the medians were calculated and compared using the nonparametric Mann–Whitney *U* test. The univariate probabilities of OS and TFS were calculated using the Kaplan–Meier estimator, and their 95% confidence intervals (CI) were constructed using arcsine-transformed intervals. The log-rank test was used to compare the probabilities of survival. A stratified univariate analysis of OS and TFS was performed using the Mantel–Haenszel test. A univariate analysis of GF was performed using the Kruskal–Wallis test. Multivariate Cox regressions were performed for the variables identified as being associated with one of the endpoints, those that were marginally significant in the univariate analyses, or those with clinical relevance (e.g., age). For all tests, the *P* values were 2-sided and statistical significance was defined as *P* < 0.05. All analyses were performed using PASW statistics version 19.0 (IBM SPSS, Inc., Chicago, IL).

An exhaustive identification of patients was attempted through the Children HSCT Study Group in a multicenter study conducted by the Pediatric Branch of the Chinese Medical Association. Between January 1998 and December 2009, a total of 50 patients with *β*-TM underwent 6-allele-matched HSCT in 5 different transplantation centers in mainland China. The patient charts were analyzed retrospectively, and the data analysis was performed in December 2015. All survivors had at least 5 years of follow-up after HSCT.

## Results

### General characteristics

**Fig. 1:**
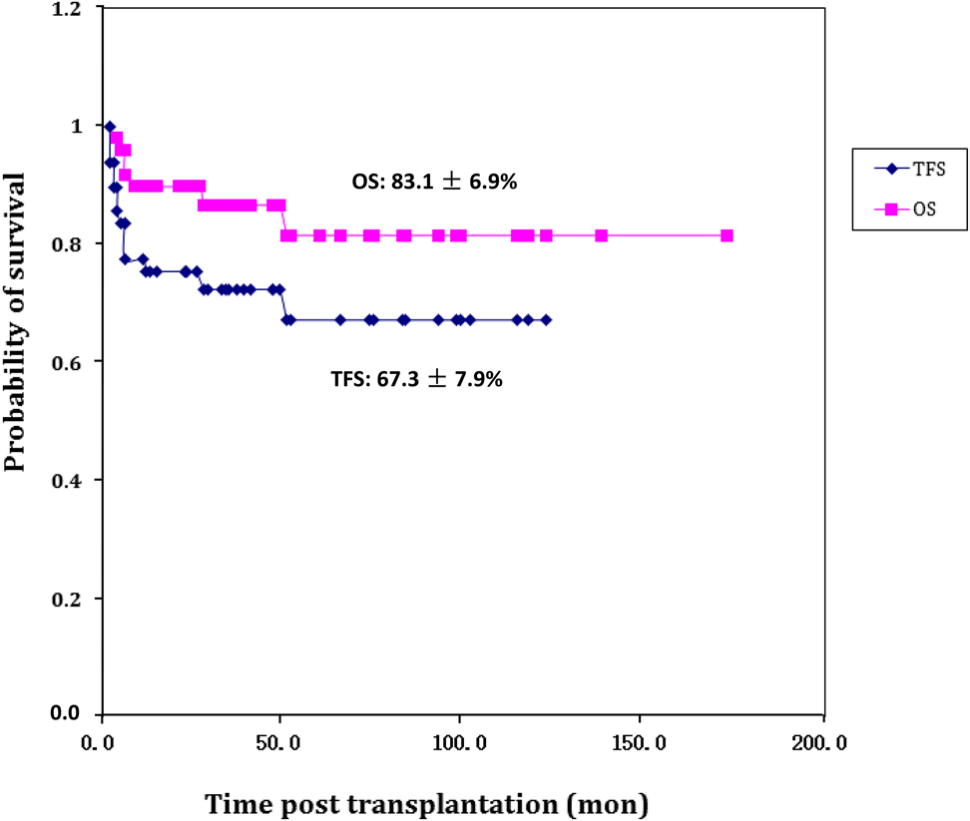
Curve of 5-year accumulated overall survival (OS) and thalassemia-free survival (TFS). The probability of 5-year accumulate OS and TFS was 83.1% (95% CI 90.0–76.2%) and 67.3% (95% CI 59.4–75.2%), respectively. *CI* confidence interval

The post-HSCT follow-ups lasted for 44.7 months on average due to mortality. The longest follow-up lasted for 11 years. Over 5 years, the probabilities of accumulated OS and TFS were 83.1 ± 6.9 and 67.3 ± 7.9%, respectively (Fig. 1). The OS pre-2001 and post-2001 were 88.9 ± 10.5 and 85.0 ± 6.4% (*P* = 0.548), respectively. The TFS pre-2001 and post-2001 were 60.0 ± 15.5 and 72.6 ± 7.6% (*P* = 0.614), respectively. Six cases (12.0%) of death were recorded: one recipient died of severe acute respiratory syndromes in the 51st month after complete engraftment, one recipient died after transplantation without engraftment, one case of death occurred due to severe GVHD with multiple organ failure after the second transplantation, and 3 patients died of severe cytomegalovirus infection or pulmonary fungal infection 6 months after successful transplantation. Both acute GVHD (aGVHD) and chronic GVHD (cGVHD) were generally controllable and not fatal. The incidence of aGVHD was 62.0% (*n* = 31), and ten of these cases were grade 2 or above. The incidence of cGVHD was 24.0% (*n* = 12). According to the Seattle clarification, 5 of the cGVHD cases were extensive and 7 were localized. All the 12 patients survived. The incidence of aGVHD pre-2001 and post-2001 was 80.0% (8/10) and 57.5% (23/40) (*P* = 0.173), respectively. The incidence of cGVHD pre-2001 and post-2001 was 30.0% (3/10) and 22.5% (9/40), respectively. The proportion of complete chimerism reached 80.0% (95% CI 71.4–89.8%), whereas the proportion of incomplete chimerism was 16.0%. When compare Pesaro class 1/2 and class 3, the proportion of complete chimerism of two groups was 82.4 and 60.0% (*P* = 0.204), respectively. There were 17 recipients with incomplete chimerism or secondary graft failure after complete chimerism, and 15 of them eventually experienced graft failure. Secondary graft rejection was the major reason for the decrease in TFS.

### Univariate analyses of OS and TFS

Univariate analyses showed that gender, UCBT (the transplanted graft contained umbilical cord blood only), the use of MTX and the use of MMF were potential key factors for OS: the impacts of donor type (related donor versus unrelated donor), graft type, conditioning regimen group and age of the recipient at transplantation (< 7 versus ≥ 7 years) on OS were not significant (*P* > 0.05). Univariate analyses also showed that graft type, UCBT and the use of MTX were potentially related to TFS; the effects of gender, donor type, conditioning regimen group, transplant age and the use of MMF on TFS were not significant. These results are displayed in Table 2.

**Table 2:** Univariate analysis of OS and TFS

Variables	OS, %	*P*	TFS, %	*P*
Gender, mean ± SE				
Male	96.2 ± 3.8	0.031	81.3 ± 7.5	0.091
Female	64.3 ± 14.7		47.7 ± 14.2	
Age at transplantation, mean ± SE				
< 7 y	89.3 ± 5.9	0.256	77.1 ± 7.7	0.103
≥ 7 y	58.3 ± 24.4		32.4 ± 23.6	
UCBT, mean ± SE				
Yes	73.3 ± 10.2	0.051	47.1 ± 11.0	0.002
No	83.3 ± 15.2		77.4 ± 14.7	
Donor type, mean ± SE				
Related	87.4 ± 7.1	0.092	70.0 ± 8.9	0.452
Unrelated	78.6 ± 11.0		66.7 ± 12.2	
Graft type, mean ± SE				
PBSC	83.3 ± 15.2	0.227	75.0 ± 15.8	0.020
UCB	73.3 ± 10.2		47.1 ± 11.0	
BM	100		100	
BM + PBSC/UCB	100		88.9 ± 10.5	
Conditioning regimen group, mean ± SE				
BUCYATG	100	0.387	70.0 ± 14.5	0.625
BUCY + X	74.9 ± 10.2		59.7 ± 11.0	
BUCYATG + Flu + Hu	92.9 ± 6.9		85.7 ± 9.4	
Use of MTX, mean ± SE				
Yes	100	0.006	92.6 ± 5.0	< 0.001
No	66.9 ± 11.4		42.1 ± 11.4	
Use of MMF, mean ± SE				
Yes	62.3 ± 15.0	0.011	53.0 ± 15.5	0.229
No	89.7 ± 7.6		71.7 ± 9.1	

*PBSC* peripheral blood stem cell, *UCB* umbilical cord blood, *BM* bone morrow, *UCBT* transplanted graft contained umbilical cord blood only, *BU* busulfan, *CY* cyclophosphamide, *ATG* anti-thymocyte globulins, *Flu* fludarabine, *Hu* hydroxyurea, *MTX* short courses of methotrexate, *MMF* mycophenolate mofetil, *BUCYATG* conditioning regimen consisting of BU, CY and ATG, *BUCYATG* *+* *Flu* *+* *Hu* conditioning regimen consisting of BU, CY, ATG, Flu, Hu and Aza, *BUCY* *+* *X* all other conditioning regimens, *use of MTX* MTX was used for the prophylaxis of graft-versus-host disease, *use of MMF* MMF was used for the prophylaxis of graft-versus-host disease, *OS* 5-year accumulated overall survival, *TFS* 5-year accumulated thalassemia-free survival, *SE* standard error

### Umbilical cord blood transplantation

UCBT for TM exhibited much lower OS (UCBT versus non-UCBT: 73.3 ± 10.2 versus 83.3 ± 15.2%, *P* = 0.051) and TFS (UCBT versus non-UCBT: 47.1 ± 11.0 versus 77.4 ± 14.7%, *P* = 0.002) than other types of grafts in the univariate analysis. Table 3 displays detailed comparisons of the recipient and donor characteristics as well as the conditioning regimens and GVHD prophylaxis between the UCBT and non-UCBT groups. UCBT used to be popular (22/50 of the cases in this study) in China. Seventeen of 35 HLA-matched HSCT from related donors involved UCBT. However, in three of these cases, engraftment failure occurred, and in five cases, recurrence occurred after engraftment. Only nine achieved continued TFS maintenance. There were only five cases of UCBT in the unrelated donor HSCT cases, and all of these achieved engraftment. However, three of these recipients died of complications, and secondary graft failure occurred in one recipient. The comparisons of GVHD incidence, GVHD severity, mortality, and study end points between the UCBT and non-UCBT groups are displayed in Table 3. Since 2005, four cases of UCB-BM combined transplantation were performed. One of these patients experienced recurrence, but the other three were persistently thalassemia free. Accordingly, the risks of mortality and recurrence decreased when PBSC or UCB were transplanted in combination with bone marrow, but this was still inferior to BMT (TFS: 88.9%, OS: 100%). A multivariate COX analysis indicated that in spite of the significantly different use of MTX between the UCBT and non-UCBT groups and tremendous difference in TFS and OS between the MTX (GVHD prophylaxis with MTX) and the non-MTX groups (GVHD prophylaxis without MTX), the use of MTX was not the key factor leading to the differences in TFS and OS between the UCBT and non-UCBT groups [relative risk (RR) = 6.265, 95% CI 0.794–49.433, *P* = 0.082]. The use of MMF was not the key factor (RR = 0.250, 95% CI 0.165–1.596, *P* = 0.515), either. Further analysis demonstrated that, in the UCBT group, related donor transplantation produced more favorable results than unrelated donor transplantation in OS (related donor versus unrelated donor: 86.2 ± 9.1 versus 25.0 ± 21.7%, *P* = 0.009) but not in TFS (related donor versus unrelated donor: 55.6 ± 12.6 versus 20.0 ± 17.9%, *P* = 0.217).

**Table 3:** Detailed comparisons between the UCBT and non-UCBT groups

Variables	UCBT	Non-UCBT	*P*
Gender, *n*			
Male	13	15	0.696
Female	9	13	
Transplantation age (y), median (range)	5.0 (1.3–12.0)	5.0 (1.0–14.0)	0.945
Donor type, *n*			
RD	17	18	0.320
UD	5	10	
Hu usage, *n*			
Yes	6	12	0.254
No	16	16	
Conditioning regimen, *n*			
BUCYATG	6	5	0.366
BUCYATG + Flu + Hu	4	10	
BUCY + X	12	13	
MTX usage, *n*			
Yes	2	26	< 0.001
No	20	2	
aGVHD incidence, *n* (%)	14 (63.6)	17 (60.7)	0.833
aGVHD grade 2-4, *n* (%)	3 (13.6)	7 (25.0)	0.325
cGVHD incidence, *n* (%)	4 (18.2)	8 (28.6)	0.393
Limited, *n* (%)	2 (9.1)	5 (17.9)	0.295
Extensive, *n* (%)	2 (9.1)	3 (10.7)	0.849
Mortality, *n* (%)	5 (26.3)	1 (3.6)	0.022
Graft failure, *n* (%)	9 (40.9)	2 (7.1)	0.004
OS, % (95% CI)	73.3 (83.5–63.1)	83.3 (98.5–68.1)	0.051
TFS, % (95% CI)	47.1 (58.1–36.1)	77.4 (92.1–62.7)	0.002

Details on recipient and donor characteristics as well as conditioning regimens and graft-versus-host disease (GVHD) prophylaxis comparisons between the umbilical cord blood transplantation (UCBT) and non-UCBT groups

*RD* related donor, *UD* unrelated donor, *BU* busulfan, *CY* cyclophosphamide, *ATG* anti-thymocyte globulins, *Flu* fludarabine, *Hu* hydroxyurea, *aGVHD* acute GVHD, *cGVHD* chronic GVHD, *BUCYATG* conditioning regimen consisting of BU, CY and ATG, *BUCYATG* *+* *Flu* *+* *Hu* conditioning regimen consisting of BU, CY, ATG, Flu, Hu and Aza, *BUCY* *+* *X* all other conditioning regimens, *OS* 5-year accumulated overall survival, *TFS* 5-year accumulated thalassemia-free survival

### Conditioning regimen groups

For the analysis of the conditioning regimens, the incidences of TFS, OS and GF were compared among 3 groups: BUCYATG (*n* = 11), BUCY + X (*n* = 25) and BUCYATG + Flu + Hu (*n* = 14). The OS of the BUCYATG BUCY + X and BUCYATG + Flu + Hu groups was 100%, 74.9 ± 10.2% and 92.9 ± 6.9%, respectively (*P* = 0.387), and the TFS of these three groups was 70.0 ± 14.5%, 59.7 ± 11.0% and 85.7 ± 9.4%, respectively *P* = 0.625). The relationship between the conditioning regimen group and GF was analyzed, and the between-group differences in the GF incidence were not significant (BUCYATG versus BUCYATG + Flu + Hu: 27.3 versus 14.3%, *P* = 0.763; BUCYATG versus BUCY + X: 27.3 versus 24.0%, *P* = 1.00; BUCY + X versus BUCYATG + Flu + Hu: 24.0 versus 14.3%, *P* = 0.759). The difference among all three groups was also insignificant (*P* = 0.723).

## Discussion

At the beginning phase of HSCT for the treatment of *β*-TM, doctors in mainland China were faced with numerous difficulties, including HLA matching techniques, the toxicity of conditioning regimens, graft rejection, severe GVHD, infections, patients’ high risk status, and a lack of appropriate donors. However, none of these challenges stopped the advancement of HSCT in mainland China. Doctors kept searching for advanced techniques to improve prognoses; for example, ATG was introduced in 48 of 50 cases and different conditioning regimens were applied.

In our study, BMT has been proven to be the safest and most efficient technique for treating *β*-TM. However, in cases of younger sibling donors and unrelated donors, the bone marrow volume harvested is usually limited. Other types of grafts and combined transplantation were considered and developed. UCBT, which used to be popular in China due to its low cost and less traumatic procedures, has been used to cure leukemia with outcomes similar to those of sibling donor transplantation, as reported previously by our study group [18]. However, the situation was exactly the opposite in *β*-TM, as shown by the results. Compared with reports from other centers, UCBT in mainland China did not provide equivalent outcomes. According to the previous reports in mainland China, most UCBT recipients achieved autologous recovery or eventually died of complications; by contrast, the implantation after PBSCT was much better [8]. Global data have suggested that UCBT from related donors had similar engraftment levels compared to BMT but lower incidences of GVHD, which further promoted the use of UCBT in treating *β*-TM [19–22]. Similar to the global experience, doctors from Taiwan District reported their experiences with UCBT in 2009, suggesting that an adequate NC count was the key to successful UCBT [23]. In 2012, researchers in Taiwan of China presented another informative single-center experience reporting that the most important risk factors for a poor prognosis were iron overload and the elevated panel-reactive antibody (PRA) caused by hypertransfusion. Thus, they recommended the use of UCBT at a young age, when the transfusion volume is not large [24]. Researchers from Europe claimed that the NC was not relevant to the outcome. UCBT from unrelated donors resulted in lower TFS (21%), whereas UCBT from matched sibling donors had a similar TFS to that of BMT [22]. However, in the UCBT group, related donor transplantation did not produce more favorable TFS results compared to unrelated donor transplantation (related donor versus unrelated donor: 55.6 ± 12.6 versus 20.0 ± 17.9%, *P* = 0.217). According to a retrospective study, graft rejection after UCBT may be related to conditioning regimens and GVHD prophylaxis. The use of MTX for GVHD prophylaxis was associated with a greater risk of treatment failure [21]. In the present study, NC and the use of MTX were controlled as recommended but graft failure was still the major cause of UCBT failure. The conditioning regimen, elevated PRA and cellular activities of UCB were presumed to be the main reasons.

Before 2000, most patients accepted whole-blood transfusions without white cell depletion, which induced elevated PRA levels and thus increased the risks of graft rejection and interfered with stem cell proliferation [25]. Furthermore, irregular and inadequate transfusions led to the expansion of bone marrow and extramedullary hematopoiesis. Hypertransfusion and the suppression of hematopoiesis have proven to be successful in improving engraftment [26, 27]. However, due to the limited blood resources in mainland China, more relatives were needed as blood donors to the recipients when hypertransfusion was required, inducing elevated minor histocompatibility antigen alloimmunization as well as rejection incidence and primary graft failure [28, 29]. Overcoming extramedullary hematopoiesis without stimulating PRA is probably the major task for controlling the incidence of complications.

In this study, the OS and TFS tended to differ among the three conditioning regimen groups, though these differences were not significant. The BUCYATG treatment was shown to be the safest, whereas the BUCY + X treatment was the least safe. The safety of the conditioning regimen of BUCYATG + Flu + Hu was between these values. However, the safest regimen was not the most successful, as the TFS of the BUCYATG group was lower than that of the BUCYATG + Flu + Hu group. The intensified conditioning regimen of BUCY + X was disappointing and was eventually eliminated, whereas the conditioning regimen of BUCYATG + Flu + Hu has become dominant in HSCT for *β*-TM over the comparably simple BUCYATG regimen.

As reported lately, the appropriately modified conditioning regimen can successfully improve the outcomes of class 3 recipients [30]. For the impaired heart and liver functions of class 3 recipients, these patients had difficulty tolerating the intensified conditioning regimen that was previously recommended [31–33]. The BUCYATG + Flu + Hu conditioning regimen has been performed since 2001. This modified protocol in the retrospective cohort has shown improvements in disease-free survival and reductions of treatment-related mortality in high-risk patients. Before transplantation, patients, especially those who tended to be high risk, strongly required regular transfusions and iron chelation, which tend to reduce extramedullary hematopoiesis. Unlike in Protocal 26 reported by Sodani et al. from Italy [26], granulocyte colony-stimulating factor, erythropoietin and growth factor were not provided to recipients due to the inconvenient procedures. By contrast, the comparably shorter courses of Hu and Aza prevented outpatient infections and severe liver damage. This new and simple conditioning regimen should be promoted, and further study should be conducted to conclusively demonstrate its efficiency and safety.

Previous studies recommended intensified conditioning regimens for Asian populations. Increasing the dose of Bu to 20 mg/kg [34] and the addition of Mel and TT [13] both helped to decrease the rejection rate. Those conditioning regimens that were not sufficiently intensified were not able to maximally eliminate marrow and extramedullary hematopoiesis, which might explain why the BUCYATG conditioning regimen did not achieve the highest TFS among the three groups. The recipient’s T lymphocytes, which may be responsible for the rejection of a donor’s grafted cells, are effectively depleted by ATG, but the value of including ATG in conditioning regimens is still controversial [17, 19, 26, 27, 35–37]. In the present study, all but two patients accepted ATG-containing conditioning regimens for the most intensive depletion of recipient T lymphocytes without the use of other T cell-depleting agents. The use of ATG in HSCT from different donors and different types of grafts should be further investigated, and designing an accurate regimen for each individual would be an important step toward successful HSCT.

In conclusion, in HLA-matched HSCT for *β*-TM performed between 1998 and 2009, probabilities of 5-year accumulated OS and TFS found in this study were 83.1 ± 6.9 and 67.3 ± 7.9%, respectively. GF was the main cause of transplantation failure, especially in UCBT. UCBT from related donors did not produce favorable TFS. For patients with *β*-TM in mainland China, a variety of factors affecting OS and TFS after HSCT seem to exist. A suitable conditioning regimen is a key factor for the success of HSCT. The modified myeloablative regimen of BUCYATG + Flu + Hu warrants further clinical study, and the combined transplantation of UCB and BM could improve the prognosis of UCBT. However, this was a non-prospective and non-randomized study, and the conditioning regimens and GVHD prophylaxis were too heterogeneous to allow robust conclusions. We strongly recommend conducting a prospective randomized clinical trial to evaluate risk factors and the new protocols. We also recommend increased attention to be directed toward the quality of life of post-HSCT patients.
